# Comparison Between Early Appendectomy vs. Conservative Management in Cases of Appendicular Mass

**DOI:** 10.7759/cureus.37986

**Published:** 2023-04-22

**Authors:** Bilal Tarar, Sadaf Batool, Shahid Majeed, Aimen Saleem

**Affiliations:** 1 General Surgery, Northwick Park Hospital, London, GBR; 2 Surgery, Chesterfield Royal Hospital, Chesterfield, GBR; 3 General Surgery, Combined Military Hospital, Lahore, PAK; 4 Paediatric Surgery, Children's Hospital and Medical Center, Lahore, PAK

**Keywords:** s: appendectomy, intestinal perforation, early appendectomy, conservative management, appendicular mass

## Abstract

Introduction: At present, the treatment of choice for appendicular masses is unclear. Recent studies claimed that conservative management of appendicular masses was safe in terms of frequency of perforation. However, there is controversy in the existing literature.

Objective: This research is designed to compare the results of early appendectomy versus conservative management of appendicular masses.

Material and methods: It was a randomized controlled trial performed in the Combined Military Hospital, Lahore. The study lasted six months, from 01/03/2019 to 30/09/2019. It involved 60 patients of both genders aged between 16 and 70 years diagnosed with appendicular masses with an Alvarado score of 4-7. These patients were randomly divided into two treatment groups. In Group A patients, an early appendectomy was performed, while patients in Group B were managed conservatively. Outcome variables were the mean length of hospital stay and frequency of appendicular perforation.

Results: The mean age of the patients was 26.8±11.9 years. There were 33 (55.0%) male and 27 (45.0%) female patients, with a male-to-female ratio of 1.2:1. The mean length of hospital stay was significantly longer in patients managed conservatively as compared to those undergoing early appendectomy (2.80±1.54 vs. 1.83±0.83; p=0.004). However, the frequency of perforation was not significantly higher in the conservative group as compared to the early appendectomy group (16.7% vs. 10.0%; p=0.448).

Conclusion: Conservative management of patients with appendicular mass was associated with prolonged hospital stays, yet it was found equally safe in terms of frequency of appendicular perforation, which advocates conservative management of patients with appendicular mass, particularly in high-risk patients.

## Introduction

The role of the appendix is not yet clear, even though its role in the immune system is strongly suggested by the presence of lymphatic tissue. The lifetime risk of developing appendicitis is 8.6% for men and 6.7% for women. The yearly incidence rate of perforated appendicitis is about two per 10,000 [1]. Acute appendicitis is sometimes contained by the body's defense mechanisms, such as the formation of an inflammatory phlegmon or a circumscribed abscess. The management of these patients is controversial [2]. The traditional treatment of appendicular lumps is conservatively followed by delayed appendectomy. During conservative treatment, 10%-20% are not resolved and lead to gangrene or perforation, followed by localized abscess or generalized peritonitis requiring early surgical intervention [3,4].

Early exploration is safe, confirms the diagnosis, removes the need for readmission, is curative, time-saving, reduces the cost of management, and shortens hospital stays with an early return to work [5]. Currently, the standard of care for uncomplicated acute appendicitis is surgical. Mason [6] performed what he described as a systematic review of the published literature to measure the need of performing surgery for appendicitis [7]. Mason’s study at least objects to the tradi­tional approach in the treatment of acute appendicitis. Randomized controlled trials by Hansson and colleagues [8] for assessing the use of antibiotic therapy versus appendectomy as the primary treatment of acute appendicitis. The efficacy of surgical treatment was established by the authors as 89.2%, while it was 90.8% for antibiotics; however, the occurrence of overall major compliance in patients who underwent surgery was three times higher than in patients treated with antibiotics (p < 0.05).

One study showed that the mean hospital stay was 2.3±0.3 days with conservative management and 1.2±2.1days with appendectomy for appendicular mass (p<0.05), but a perforated appendix was observed in 5% of both groups (p>0.05) [6]. But Eriksson et al. found that the mean hospital stay was 3.1±0.3 days with conservative management and 3.4±1.9 days with appendectomy for appendicular mass (p>0.05), while perforated appendix occurred in 5% of patients with surgery and 10% with conservative management [9]. This was also supported by Hansson et al. [8], who also showed that the mean hospital stay was equal in both groups, i.e., 3±0.1 with conservative management and 3±0.3 with surgery (p>0.05) [8]. In some retrospective studies, while making comparisons between “expectant” and “aggressive” management of suspected appendicitis patients, in the group of expectant management patients, only a few patients needed appendicectomy [10].

A literature review shows controversial results regarding early versus delayed appendicectomy in appendicular masses. This study aims to compare the outcome of early appendectomy with conservative management in this subgroup of patients presenting to a tertiary care hospital.

## Materials and methods

It is a randomized controlled trial. The research was performed in the surgical unit of CMH Lahore. The study lasted six months, from 01/03/2019 to 30/09/2019. A sample size of 60 cases (30 cases in each group) was calculated with a 95% confidence interval. Patients were selected by non-probability, consecutive sampling. 

Appendicular mass was defined as the presence of inflammation in acute appendicitis enclosed by defense mechanisms by the formation of an inflammatory phlegmon or a circumscribed abscess on abdominal ultrasound and an Alvarado score of 4-7. The outcome was assessed in terms of the following: A perforated appendix was labeled if perforation was directly visualized postoperatively in the early appendectomy group. In the conservative treatment group, spreading pain from the right iliac fossa to involve the generalized abdomen and tenderness in the whole abdomen was labeled as a perforated appendix. Mean hospital stay was measured in terms of the number of days required to stay in the hospital after inclusion. The patient was discharged when he/she was able to walk or go to the washroom himself/herself, could take oral feed or medicine, and had no pain on the site of the appendix. 

Inclusion criteria were patients of the age range 16-70 years of either gender presenting with appendicular mass (as per the operational definition). Patients with ASA III and IV, abdominal malignancy, and a previous history of failed medical management within six months for appendicitis were excluded from the study. 

After receiving approval from the hospital's ethical committee, 60 patients (30 in each group) fulfilling the selection criteria were enrolled in the study through the emergency department of surgery. Demographics, including name, age, gender, duration of symptoms, Alvarado score, and hospital stay, were recorded. Then all patients were randomly allocated into two equal groups using the lottery method. In group A, an appendectomy was performed. In group B, patients were managed conservatively, including 500mg of ampicillin, 80mg of gentamycin, and 500mg of metronidazole for standard weight regimens. During surgery in group A or during follow-up in group B in six weeks, the appendix was assessed to see if it was perforated. All surgeries in Group A were done by a single senior surgeon with at least four years of residency experience. All patients were followed up till discharge. 

The Data was analyzed using IBM Corp. Released 2011. IBM SPSS Statistics for Windows, Version 20.0. Armonk, NY: IBM Corp. Mean ± S.D has been given for quantitative variables, while frequency and percentage have been given for qualitative variables like gender and perforated appendix. Both groups have been compared for mean hospital stay by using independent sample t-tests for continuous variables and by using chi-square test for categorical variables. A p-value of ≤0.05 was considered significant. Data has been stratified for age, gender, Alvarado score, and duration of symptoms. Post-stratification, tests of significance have been applied to compare both groups for outcome in each stratum.

## Results

The mean age of the study population was 26.8±11.9 years. A significant proportion (n=51, 85.0%) of the patients were aged under 35 years. There were 33 (55.0%) male and 27 (45.0%) female patients, with a male-to-female ratio of 1.2:1. The Alvarado score ranged from 4 to 7, with a mean of 5.4±1.1, while the duration of symptoms ranged from 1 day to 13 days, with a mean of 7.1±3.2 days. These findings have been summarized in Table 1. 

**Table 1:** Baseline Characteristics of Study Population

Characteristics	Participants n=60
Age (years)	26.8±11.9
<35 years	51 (85.0%)
≥35 years	9 (15.0%)
Gender	
Male	33 (55.0%)
Female	27 (45.0%)
Alvarado Score	5.4±1.1
4-5	31 (51.7%)
6-7	29 (48.3%)
Duration of Symptoms (days)	7.1±3.2
1-6 days	25 (41.7%)
7-13 days	35 (58.3%)

There was no statistically significant difference between the two groups in terms of mean age (p=0.940), mean Alvarado score (p=0.727), mean duration of symptoms (p=0.691), and distribution of various subgroups based on age (p=0.718), gender (p=0.795), Alvarado score (p=0.796), and duration of symptoms (p=0.793), as shown in Table 2. 

**Table 2:** Baseline Characteristics of Study Groups n=60. Chi-square test, independent sample t-test, observed difference was statistically insignificant.

Characteristics	Conservative Management n=30	Early Appendectomy n=30	P-value
Age (years)	26.7±11.5	26.9±12.5	0.940
<35 years	26 (86.7%)	25 (83.3%)	0.718
≥35 years	4 (13.3%)	5 (16.7%)	
Gender			
Male	17 (56.7%)	16 (53.3%)	0.795
Female	13 (43.3%)	14 (46.7%)	
Alvarado Score	5.5±1.0	5.4±1.2	0.727
4-5	15 (50.0%)	16 (53.3%)	0.796
6-7	15 (50.0%)	14 (46.7%)	
Duration of Symptoms (days)	7.3±3.6	6.9±2.9	0.691
1-6 days	12 (40.0%)	13 (43.3%)	0.793
7-13 days	18 (60.0%)	17 (56.7%)	

The mean length of hospital stay was significantly longer in patients managed conservatively as compared to those undergoing early appendectomy (2.80±1.54 vs. 1.83±0.83; p=0.004), as shown in Table 3. However, the frequency of perforation was only insignificantly higher in the conservative group as compared to early appendectomy (16.7% vs. 10.0%; p=0.448), as shown in Table 4. Similar differences were noted between the groups across various subgroups based on the patient’s age, gender, Alvarado score, and duration of symptoms, as shown in Tables 5, 6.

**Table 3:** Comparison of Mean Length of Hospital Stay (Days) Between the Study Groups n=60. Independent sample t-test, * Observed difference was statistically significant

Characteristics	Conservative Management n=30	Early Appendectomy n=30	P-value
Mean Length of Hospital Stay (days)	2.80±1.54	1.83±0.83	0.004*

**Table 4:** Comparison of Appendicular Perforation Between the Study Groups n=60. Chi-square test, observed difference was statistically insignificant

Perforated Appendix	Conservative Management n=30	Early Appendectomy n=30	P-value
Yes	5 (16.7%)	3 (10.0%)	0.448
No	25 (83.3%)	27 (90.0%)	
Total	30 (100.0%)	30 (100.0%)	

**Table 5:** Comparison of Mean Length of Hospital Stay (Days) Between the Study Groups Across Various Subgroups n=60. Independent sample t-test, * Observed difference was statistically significant

Subgroups	Mean Length of Hospital Stay (days)		P-value
	Conservative Management n=30	Early Appendectomy n=30	
Age			
<35 years	2.81±1.55	1.84±0.85	0.008*
≥35 years	2.75±1.71	1.80±0.84	0.307
Gender			
Male	2.76±1.68	1.81±0.83	0.050*
Female	2.85±1.41	1.86±0.86	0.036*
Alvarado Score			
4-5	2.60±1.40	1.75±0.86	0.050*
6-7	3.00±1.69	1.93±0.83	0.041*
Duration of Symptoms			
1-6 days	2.67±1.56	1.69±0.75	0.055
7-13 days	2.89±1.57	1.94±0.90	0.037*

**Table 6:** Comparison of Appendicular Perforation Between the Study Groups Across Various Subgroups n=60. Chi-square test, observed difference was statistically insignificant

Subgroups	Appendicular Perforation (%)		P-value
	Conservative Management n=30	Early Appendectomy n=30	
Age			
<35 years	4/26 (15.4%)	3/25 (12.0%)	0.725
≥35 years	1/4 (25.0%)	0/5 (0.0%)	0.236
Gender			
Male	3/17 (17.6%)	2/16 (12.5%)	0.680
Female	2/13 (15.4%)	1/14 (7.1%)	0.496
Alvarado Score			
4-5	2/15 (13.3%)	1/16 (6.3%)	0.505
6-7	3/15 (20.0%)	2/14 (14.3%)	0.684
Duration of Symptoms			
1-6 days	2/12 (16.7%)	1/13 (7.7%)	0.490
7-13 days	3/18 (16.7%)	2/17 (11.8%)	0.679

## Discussion

With time, the forecasting and best treatment strategies for appendicitis have changed. Surgery was once believed to be the only management option for acute appendicitis [2,3]; however, it has been lately suggested that it can be treated non-surgically with antibiotics [6,8,11,12]. There is some evidence to suggest interval appendectomy to reduce the likelihood of recurrent appendicitis and of a missed neoplasm; despite that, there seems to be a growing tendency toward the use of antibiotics only and circumvention of surgery overall [13-15]. The patient may then have a radiologic or endoscopic examination to rule out an overlooked cancerous lesion. In a recent paper, St. Peter and colleagues [15] examined complicated appendicitis in children and observed that interval appendec­tomy with initial percutaneous drainage of an abscess, where possible, showed similar results to initial appendec­tomy. It was demonstrated by Marin and colleagues [16] that in managing complicated appendicitis, it is effective and safe to use percutaneous drainage. 

In the present study, the mean age of the patients was 26.8±11.9 years. A similar mean age among patients with acute appendicitis was previously reported by Rather et al. [17] in 2013 (26±11 years) in India and Al-Shahwany et al. [18] in 2012 (27±12 years) in Iraq. 

In our study, there were 33 (55.0%) male and 27 (45.0%) female patients. Male predominance was also observed by Memon et al. [19] in 2009 (65% vs. 35%). Jalil et al. [20] in 2011 (58% vs. 42%), Soomro et al. [21] in 2008 (66.07% vs. 33.92%), and Memon et al. [22] in 2013 (71.8% vs. 28.2%) in the local population, Talukder et al. [23] (58% vs. 42%) in Bangladesh, Beek et al. [24] (53% vs. 47%) in the Netherlands, and Pogorelić et al. [25] (55.3% vs. 44.7%) in Europe also found a similar trend.

Our study demonstrated that the mean length of hospital stay was significantly longer in patients managed conservatively as compared to those undergoing early appendectomy (2.80±1.54 vs. 1.83±0.83; p=0.004). However, the frequency of perforation was only insignificantly higher in the conservative group as compared to early appendectomy (16.7% vs. 10.0%; p=0.448). It was also noticed by Malik et al. [26], who reported a significant difference in the mean length of hospital stay with conservative management (2.3±0.3 vs. 1.2±2.1 days; p<0.05) of acute appendicitis without any significant difference in the frequency of appendicular perforation (5.0% vs. 5.0%; p=1.000). Styrud et al. [27] also observed a similarly prolonged duration of hospital stay with conservative management (3.0±1.4 vs. 2.6±1.2 days; p<0.05) as compared to early appendectomy. They, too, reported an insignificant difference in the frequency of appendicular perforation (5.5% vs. 4.0%; p>0.005) between the groups. A similar but insignificantly higher frequency of appendicular perforation has been reported by Eriksson et al. [9] (10.0% vs. 5.0%; p>0.05) with conservative management versus early appendectomy. 

The present study is unique in the local population and contributes to the limited research evidence on the topic from this part of the world. The strengths of our study are the randomization of the study sample and the post-stratification comparison of groups to address effect modifiers. However, our study was limited by the fact that we didn’t compare these two groups in terms of complications like peritonitis and recurrent attacks of acute appendicitis, which required longer follow-up. However, such a study is required and recommended to further establish the safety of this alternative approach before adopting it routinely. 

## Conclusions

Conservative management of patients with appendicular mass was associated with prolonged hospital stays, yet it was found equally safe in terms of frequency of appendicular perforation, which advocates conservative management of patients with appendicular mass in future practice to avoid unnecessary surgical intervention and associated complications of anesthesia and surgery, particularly in high-risk patients.
